# The Predictive Performance and Stability of Six Species Distribution Models

**DOI:** 10.1371/journal.pone.0112764

**Published:** 2014-11-10

**Authors:** Ren-Yan Duan, Xiao-Quan Kong, Min-Yi Huang, Wei-Yi Fan, Zhi-Gao Wang

**Affiliations:** 1 School of Life Science, Anqing Normal College, Anqing, Anhui, PR China; 2 College of Life Science, Shaanxi Normal University, Xi’an, Shaanxi, PR China; National Institute of Genomic Medicine, Mexico

## Abstract

**Background:**

Predicting species’ potential geographical range by species distribution models (SDMs) is central to understand their ecological requirements. However, the effects of using different modeling techniques need further investigation. In order to improve the prediction effect, we need to assess the predictive performance and stability of different SDMs.

**Methodology:**

We collected the distribution data of five common tree species (*Pinus massoniana*, *Betula platyphylla*, *Quercus wutaishanica*, *Quercus mongolica* and *Quercus variabilis*) and simulated their potential distribution area using 13 environmental variables and six widely used SDMs: BIOCLIM, DOMAIN, MAHAL, RF, MAXENT, and SVM. Each model run was repeated 100 times (trials). We compared the predictive performance by testing the consistency between observations and simulated distributions and assessed the stability by the standard deviation, coefficient of variation, and the 99% confidence interval of Kappa and AUC values.

**Results:**

The mean values of AUC and Kappa from MAHAL, RF, MAXENT, and SVM trials were similar and significantly higher than those from BIOCLIM and DOMAIN trials (*p*<0.05), while the associated standard deviations and coefficients of variation were larger for BIOCLIM and DOMAIN trials (*p*<0.05), and the 99% confidence intervals for AUC and Kappa values were narrower for MAHAL, RF, MAXENT, and SVM. Compared to BIOCLIM and DOMAIN, other SDMs (MAHAL, RF, MAXENT, and SVM) had higher prediction accuracy, smaller confidence intervals, and were more stable and less affected by the random variable (randomly selected pseudo-absence points).

**Conclusions:**

According to the prediction performance and stability of SDMs, we can divide these six SDMs into two categories: a high performance and stability group including MAHAL, RF, MAXENT, and SVM, and a low performance and stability group consisting of BIOCLIM, and DOMAIN. We highlight that choosing appropriate SDMs to address a specific problem is an important part of the modeling process.

## Introduction

Species distribution models (SDMs), also known as climate envelope models, habitat suitability models, and ecological niche models, use environment data for sites of occurrence (presence) of a species to predict a response variable, such as suitability, for a site where the environmental conditions are suitable for that species to persist and so may be expected to occur [1]–[7]. Since Nix et al. begin to model the distribution of crop species in Australia [8], empirical SDMs have emerged as valuable tools for predicting both animal and plant distributional patterns [9], [10]. Interest in SDMs has increased dramatically, motivated principally by the need to solve current ecological problems, such as understanding the ecological requirements of species [11], assessing biodiversity [12], managing nature reserves [1], predicting the potential for invasion [13], and modeling biological responses to climate change [3], [6].

A variety of SDMs, each with specific advantages and disadvantages, have been introduced to predict species spatial distribution [14]. Differences in predictive performance arise from different construction principles, the selection of environment variables considered in the model, the specific distribution areas and data input requirements (presence/absence data (*PA*) and presence-only data (*PO*)) [2], [4], [5], [7], [15]–[18]. There are substantial discrepancies in predicting species’ distributions by SDMs with different predictive modeling method, which have highlighted the uncertainties of prediction results [14], [19]–[21]. These uncertainties of prediction may puzzle stakeholders and policy makers, and cast doubt on the reliability of species distribution predictions by SDMs. Therefore, critical assessment of the predictive performance and stability of SDMs need to be performed.

Model prediction performance can be assessed by Kappa statistic, area under the receiver operating characteristic curve (AUC), overall accuracy, sensitivity, specificity, and true skill statistic (TSS). Among them, the Kappa and AUC are most often used [22], [23]. In contrast to predictive accuracy, such studies seldom consider the stability of SDMs as revealed by the distribution of Kappa and AUC values after multiple trials [24]. Our aim here is to assess the predictive performance and stability of different modeling techniques by evaluating the Kappa and AUC values. To achieve this, we selected five common tree species (*Pinus massoniana*, *Betula platyphylla*, *Quercus wutaishanica*, *Quercus mongolica* and *Quercus variabilis*) in China to evaluate six widely used species distribution models, BIOCLIM, DOMAIN, MAHAL (Mahalanobis distance), RF (random forests), MAXENT (maximum entropy) and SVM (support vector machine). We highlight that there are significant differences in prediction performance and stability among different models.

## Materials and Methods

### Data description

One coniferous species *(P. massoniana*) and four broad-leaf species (*B. platyphylla*, *Q. wutaishanica*, *Q. mongolica* and *Q. variabilis*) were chosen as test species. They are all common and dominant species widely distributed in China. Original distribution data come from the Eco-Environmental Sciences Research Center in China. The data are based on the vegetation surveys and research results in ecological system evaluation and ecological function regionalization. The data are presence-only. Distribution data were gathered from the Database for Ecosystems and Ecosystem Service Zoning in China (http://www.ecosystem.csdb.cn). All species distribution maps were rasterized at a spatial resolution of five arc-minutes. Finally we obtained 312 presence points for *Q. variabilis*, 1421 points for *B. platyphylla*, 2572 points for *Q. mongolica*, 256 points for *Q. wutaishanica*, and 4079 points for *P. massoniana*.

Building species distribution models requires accurate assessment of species distribution data. The two predominant types of data used are *PA* data and *PO* data [2], [15]–[18]. In most studies, *PO* data are obtained. In this case (only *PO* data obtained), for SDMs using *PA* data, randomly selecting pseudo-absence data (background data) are required to build and assess the model, and the pseudo-absence data selected randomly are acted as absence data [25]. So SDMs using *PA* data can be used in this way. In our studies, we selected some points as pseudo-absences data for RF and SVM.

Five hundred pseudo-absence points for every species were randomly generated from all points in the China excluding available presence points. The dataset were partitioned randomly into a training set and a test set with a ratio of 4∶1. The former was used for training the studied model for prediction, and the latter was used for testing the final model for predictive performance and stability. This process was repeated for 100 times. In each iteration, test statistics (AUC and kappa) were calculated.

### Environmental data

We collected 26 ecological-environmental variables (Table 1). Data for 19 bio-climatic factors and a geographical factor were extracted from the Global Climate Data (http://wwww.worldclim.org), representing the period 1950–2000 (i.e., the present). These data layers were produced by interpolating the average monthly climate data on a 30-arc-second (approximately 1 km^2^) resolution grid [26]. Three human disturbance factors come from the Center for International Earth Science Information Network (http://sedac.ciesin.columbia.edu), and three soil factors come from the Atlas of the Biosphere (http://wwww.sage.wisc.edu/atlas/, the Nelson Institute Center for Sustainability and the Global Environment, University of Wisconsin-Madison).

**Table 1:** Lists of the 26 environment variables.

Variable	Symbol
Annual mean temperature (°C)^a^^,^ b	Bio1
Mean diurnal range (Mean of monthly (max temp - min temp)) (°C)^a^^,^ b	Bio2
Isothermality (×100)^b^	Bio3
Temperature seasonality (standard deviation×100) (°C)^a^^,^ b	Bio4
Max temperature of warmest month (°C)^b^	Bio5
Min temperature of coldest month (°C)^b^	Bio6
Temperature annual range (°C)^b^	Bio7
Mean temperature of wettest quarter (°C)^a^^,^ b	Bio8
Mean temperature of driest quarter (°C)^b^	Bio9
Mean temperature of warmest quarter (°C)^b^	Bio10
Mean temperature of coldest of quarter (°C)^b^	Bio11
Annual precipitation (mm)^a^^,^ b	Bio12
Precipitation of wettest month (mm)^b^	Bio13
Precipitation of driest month (mm)^b^	Bio14
Precipitation seasonality (coefficient of variation) (mm)^a^^,^ b	Bio15
Precipitation of wettest quarter (mm)^b^	Bio16
Precipitation of driest quarter (mm)^b^	Bio17
Precipitation of warmest quarter (mm)^b^	Bio18
Precipitation of coldest quarter (mm)^a^^,^ b	Bio19
Human footprint^a^^,^ c	HF
Human influence index^c^	HII
Human population density in year 2000 (persons/km^2^)^a^^,^ c	HPD
Soil organic carbon density (kg/m^2^ at 1 m depth)^a^^,^ d	SOC
Soil pH value^a^^,^ d	SPH
Soil moisture index^a^^,^ d	SMI
Altitude (m)^a^^,^ b	ALT

a Variables used in modeling.

b See http://www.worldclim.org/.

c See http://sedac.ciesin.columbia.edu/.

d See http://www.sage.wisc.edu/atlas/maps.php.

Human footprint (HF) is based on the premise that the impact of human influence varies by biogeography and HF expresses as a percentage the relative human influence in every biome on the land’s surface.

Human influence index (HII) is a measure showing direct human influence on ecosystems using eight measures of human presence (population density/km^2^, score of railroads, score of major roads, score of navigable rivers, score of coastlines, score of nighttime stable lights values, urban polygons, and land cover categories).

Soil moisture index (SMI) reflects the ability of soil to supply moisture to plants and SMI can identify a quick onset of drought by demonstrating the observed dryness of a soil relative to the plant’s ability to extract water as scaled over the range from field capacity to wilting point.

The 26 variables can capture the main environmental gradients of China [27]. Previous studies have confirmed these variables are important to determine the plant distribution ranges in China [28], and some studies on species distribution range have used the similar variables as predictors of potential distribution patterns [29]–[35].

Species distribution models with many potentially relevant variables may lead to over-fitting and poor prediction performance [27], [36], [37]. Thus, prior to model building, Pearson’s correlation coefficients (*Rs*) were calculated between pairs of variables at all points in China to determine which variables to include (Appendix Figure S1 and Table S1). Variables showing a correlation (*Rs*) >0.85 were considered redundant. Between any redundant variables, only one would be kept, and others would be removed. Many previous applications of SDMs have used the similar method to choose suitable environmental variables [27], [31], [34], [36], [37]. The 13 final environmental variables were chosen as the variables parameters of SDMs (Table 1).

### Modeling algorithms

We chose six species distribution models: BIOCLIM, DOMAIN, MAHAL (Mahalanobis distance), RF (random forests), MAXENT (maximum entropy), and SVM (support vector machine). These six SDMs are widely used in academic research and species conservation [16], [17], [33], [34].

The BIOCLIM model uses environmental data of all known species distribution points and can determine the range of weather conditions suitable for species occurrence. The percentile distribution of every climatic variable within each grid in the species distributions zone is used for multivariate analysis. If the ranges of all climatic variables in the grid are within boundaries appropriate for that species, the BIOCLIM model indicates that this place is suitable [8], [17].

The DOMAIN uses a point-to-point similarity metric based on the Gower distance, which is a method for creating a distance matrix from a set of characteristics of species. DOMAIN can assign a classification value of habitat suitability index to each potential site based on its proximity in environmental space to the most similar positive occurrence location [38]. Then, a threshold value of suitability is chosen to determine the distribution boundaries of species’ ecological niche.

The MAHAL model is based on Mahalanobis distance (MD). MD considers the variables correlations in the data set without depending on the scale of measurements. The method ranks the potential sites through their MD to a vector, which can express the mean environmental values of all recorded environmental factors. A certain distance threshold can act as the ecological niche boundaries. These algorithm generate an elliptic envelope which can explicitly explain the possible interrelations between these environmental factors [21].

The RF, a classification and regression tree model, is a combination of tree predictors where every tree can depend on the values of a random vector sampled independently with the same distribution for all trees in the forest [39], [40].

MAXENT is based on a machine learning algorithm called maximum entropy, and is based on the principle that species without ecological constraints will spread as far as possible with a distribution as close as possible to uniform [41].

The SVM is a machine-learning method that belongs to a family of generalized linear classifiers. The principle of SVM is the Vapnik Chervonenkis (VC) dimension and structural risk minimization theory [42]. The SVM model can find the most reasonable way between species adaptability and complexity to yield the most likely distribution according to the limited sample information [43].

Each of the SDMs was operated with strictly following the modeling technique and using the same 13 environmental variables. Modeling data, advantages and disadvantages were listed in Table S2. We chose “R” as the computing platform and the dismo package to simulate species distribution [44], [45].

### Model evaluation and comparison

Kappa statistic and AUC were considered to be the best evaluation standard and they were widely used in SDMs [5], [17], [22], [23], [27], [31], [34], [46], [47]. We calculated Kappa and AUC values according to the methods of Fielding & Bell [46] and Hanley & McNeil [47], respectively.

The Kappa statistic for agreement is based on the optimal threshold that can make the best of the information in the mixed matrix to measure the performance of the model. Evaluation criteria for the Kappa statistic are as follows: excellent (0.85–1.0), very good (0.7–0.85), good (0.55–0.7), fair (0.4–0.55), and fail (<0.4). The AUC derived from signal detection theory is the area under the receiver operating characteristic curve (ROC). Evaluation criteria for the AUC statistic are as follows: excellent (0.90–1.00), very good (0.8–0.9), good (0.7–0.8), fair (0.6–0.7), and poor (0.5–0.6) [22], [23].

To evaluate the stability of the six models, we used the standard deviation, coefficient of variation, and 99% confidence interval of the Kappa and AUC values to reflect the scatter of results from 100 repetitions for each species. These criteria are statistically significant and widely used in statistic [48], and many previous studies have used them as stability indicators [49]–[51]. For example, Elith et al. [50] review the aspects of uncertainty in predictions of species distribution, and suggest some methods (especially the confidence intervals) for investigating and communicating these uncertainties. Olden et al. [51] use the confidence intervals to estimate the percent correct classification of different predictive models of fish species distributions.

### Predictive maps

**Figure 1:**
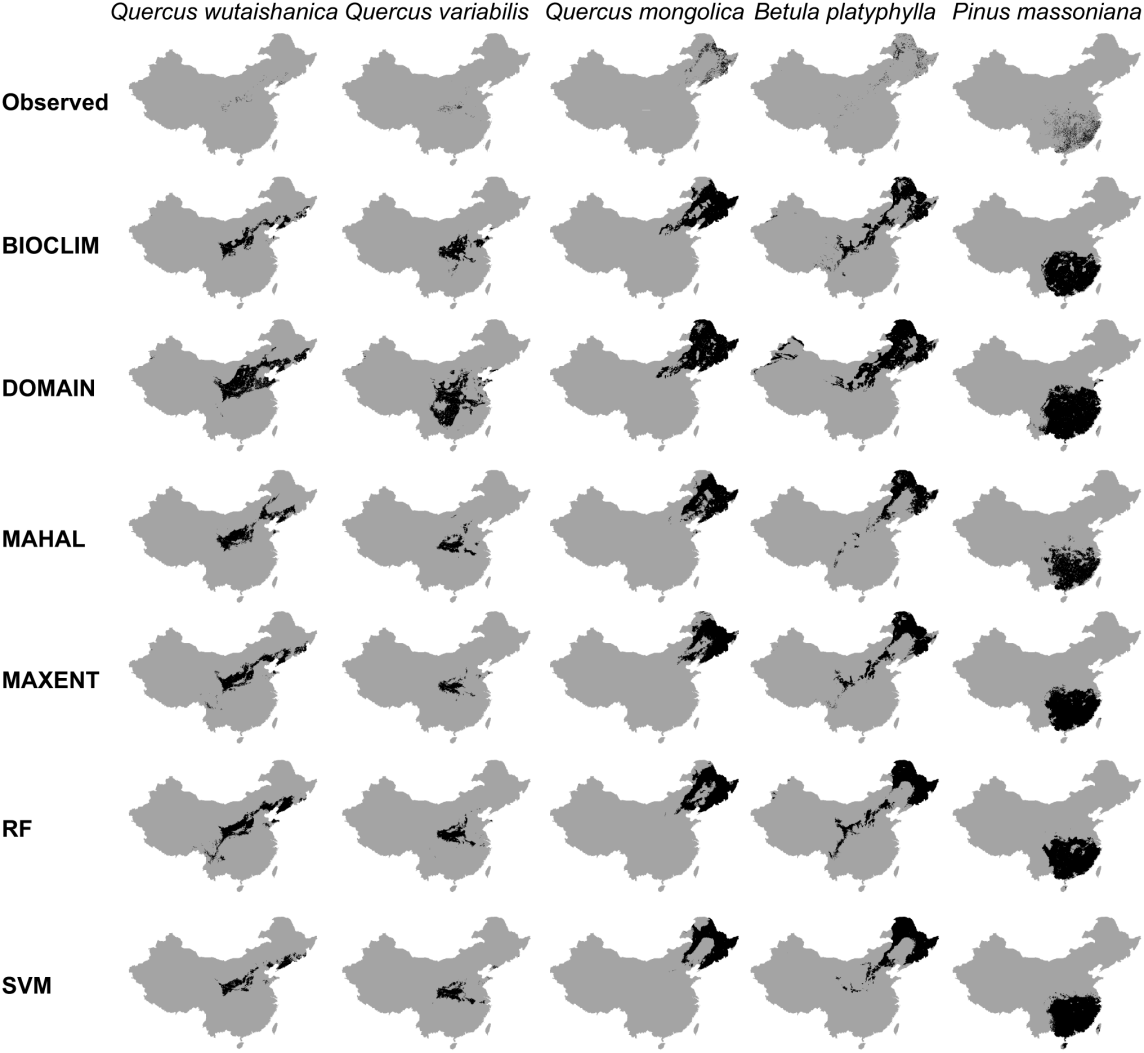
Predicted geographic distribution areas for each species (*Quercus variabilis*, *Betula platyphylla*, *Quercus mongolica*, *Quercus wutaishanica* and *Pinus massoniana*) in six SDMs (BIOCLIM, DOMAIN, MAHAL, RF, MAXENT, and SVM).

The calculated species distributions maps obtained by these models are not binary (0 or 1) or discrete data sets, but a set of continuous probability values. To determine the species distribution range, we need to set a suitable threshold value below which a species is considered absent. There are many threshold optimization criteria, such as Max of Kappa, Max (sensitivity + specificity), traditional default threshold 0.5, and so on. The threshold selection of Max (sensitivity + specificity) can minimize the mean value of the error rate for positive observation values and negative observation values. Max (sensitivity + specificity) equals to the optimal threshold point on the ROC curve whose tangent has a corresponding slope [52] and it also equals to Max (sensitivity + specificity−1), otherwise known as TSS [53]. We used the threshold value of Max (sensitivity + specificity) to generate a binary map depicting predicted areas for each species (Figure 1).

## Results

**Figure 2:**
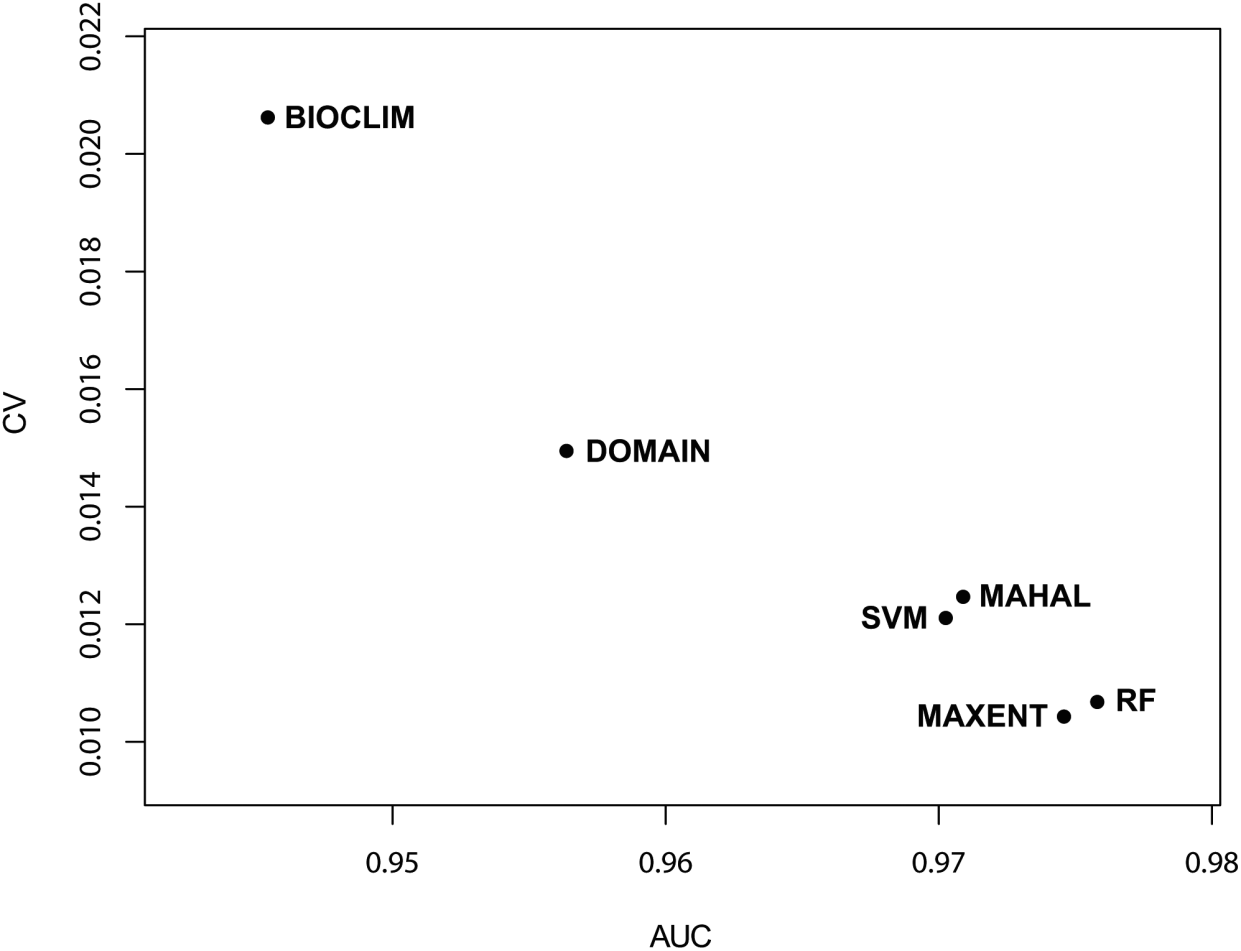
The variable coefficient (CV) of AUC for six SDMs (BIOCLIM, DOMAIN, MAHAL, RF, MAXENT, and SVM).

**Figure 3:**
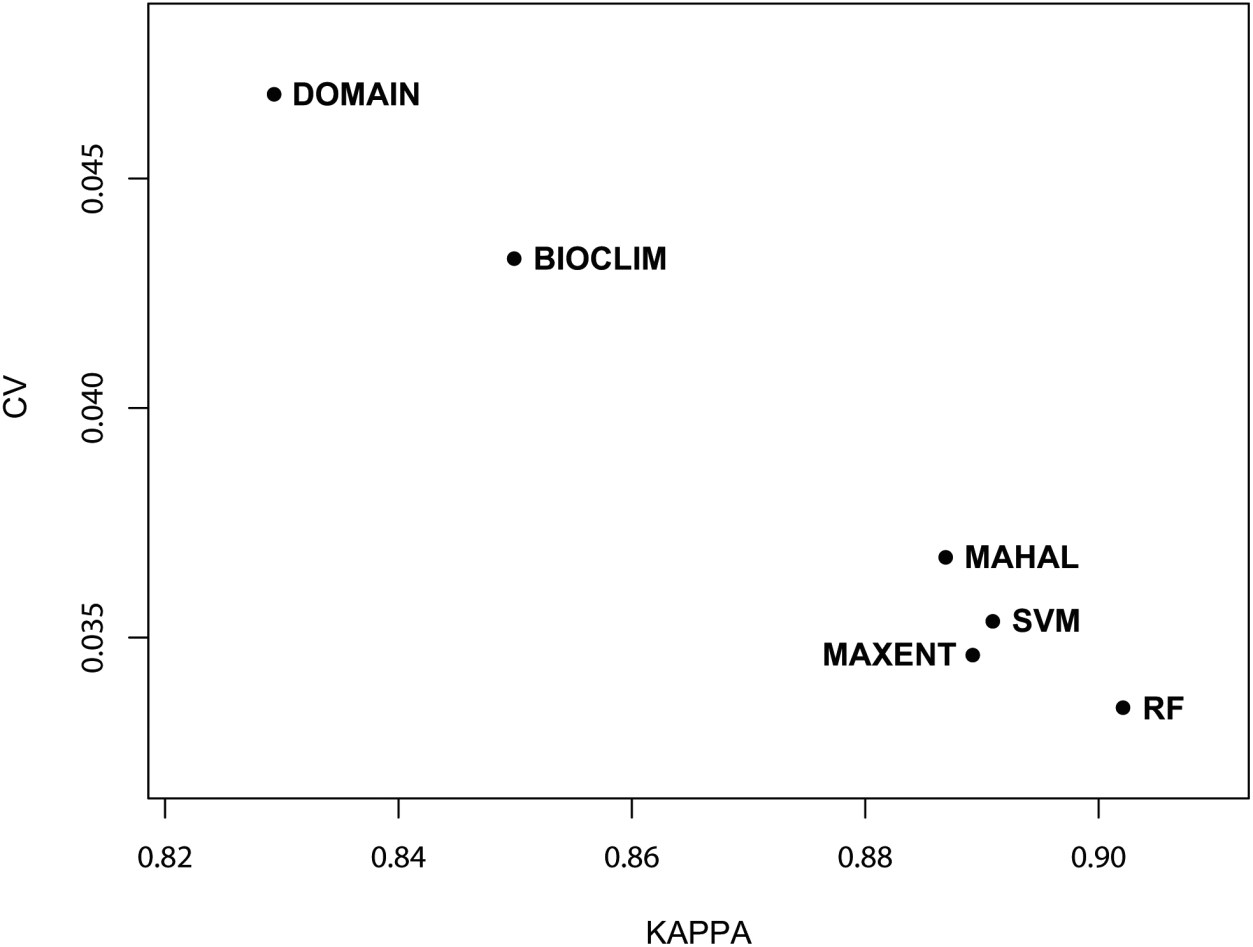
The variable coefficient (CV) of Kappa for six SDMs (BIOCLIM, DOMAIN, MAHAL, RF, MAXENT, and SVM).

To display mapped model results for the five tree species, we chose the map which was closest to the mean values of AUC for each species as the finally binary map (Figure 1 and Table S3). According to the mean values of Kappa and AUC, the prediction results were most reliable for *Q. variabilis* and least reliable for *P. massoniana.* The six SDMs were able to well predict all species distributions, though the predict results were different. The mean AUC values from 100 repetitions of the MAHAL, RF, MAXENT, and SVM models were similar (range, 0.970−0.976) and all were significantly higher (*p*<0.05) than the mean AUCs for BIOCLIM and DOMAIN (0.945 and 0.956, respectively) (Table 2). Like the mean AUC values, the mean Kappa values from MAHAL, RF, MAXENT, and SVM trials (range, 0.887−0.902) were similar and significantly higher (*p*<0.05) than the mean Kappa values for BIOCLIM and DOMAIN (0.850 and 0.829, respectively) (Table 2). The mean standard deviations (SD) of the Kappa and AUC values from BIOCLIM and DOMAIN trials were significantly higher than those for MAHAL, RF, MAXENT, and SVM (both p<0.05) (Table 2), while the 99% confidence intervals (CIs) for MAHAL, RF, MAXENT, and SVM (AUC range, 0.967−0.978; Kappa range, 0.879−0.910) were significantly narrower than those for BIOCLIM and DOMAIN (AUC, 0.940−0.960; Kappa, 0.819−0.859) (*p*<0.05 for all) (Table 2). Finally, the coefficient of variability (CV) of the mean AUC and Kappa values for BIOCLIM and DOMAIN were significantly higher than those for MAHAL, RF, MAXENT, and SVM (*p*<0.05 for both) (Figure 2 and Figure 3).

**Table 2:** The mean value and confidence interval of AUC and Kappa.

	AUC(Mean±SD)	Kappa(Mean±SD)	Confidence interval of AUC(99% confidence level)	Confidence interval of Kappa(99% confidence level)
BIOCLIM	0.945±0.019 b	0.850±0.037 b	0.940−0.950	0.840−0.859
DOMAIN	0.956±0.014 b	0.829±0.039 b	0.953−0.960	0.819−0.839
MAHAL	0.971±0.012 a	0.887±0.033 a	0.968−0.974	0.879−0.895
RF	0.976±0.010 a	0.902±0.030 a	0.973−0.978	0.894−0.910
MAXENT	0.975±0.010 a	0.889±0.031 a	0.972−0.977	0.881−0.897
SVM	0.970±0.012 a	0.891±0.031 a	0.967−0.973	0.883−0.899

Means with different letters differ significantly among the six SDMs (BIOCLIM, DOMAIN, MAHAL, RF, MAXENT, and SVM). SD is the abbreviation for standard deviation.

## Discussion

Previous studies have doubted the usefulness of SDMs method [54], [55] for their potential error sources, such as biotic errors and algorithmic errors [2]. Biotic errors can be caused by some ecological factors (e.g., biotic interactions, species dispersal ability, and population’s adaptation) that are not included in building SDMs, which can cause the prediction values of species distribution zone to depart from equilibrium assumption inhered in SDMs [56]. Algorithmic errors can be caused by the limitation of modeling techniques and the uncertainty of models that we have discussed here. Though there are some uncertainties, many studies have documented that SDMs provide the best available method to predict species’ potential ranges [14], [19]–[21]. Our results highlight there ought to be more testing and improving the method to enhance confidence in the prediction ability and stability of SDMs. By demonstrating large differences of predictive performance and stability of different modeling techniques, our study confirms that choosing appropriate SDMs to address a specific problem is an important step in the modeling process.

The six SDMs tested yielded different distribution maps using the same 13 ecological variables, and they could be divided into a high performance group (MAHAL, RF, MAXENT and SVM), and a low performance group (BIOCLIM and DOMAIN), based on their predictive performance and stability. Our result agrees well with other studies. For example, Reiss et al. [35] show that MAXENT, RF and SVM have similar predictive performance, and their AUC values are significantly higher than BIOCLIM. Tsoar et al. [17] confirm that MAHAL can predict better than BIOCLIM and DOMAIN. Elith & Graham et al. [16] show that MAXENT has significantly higher predictive performance than BIOCLIM and DOMAIN. Giovanelli et al. [34] confirm that MAXENT and SVM have a similar predictive performance, and they are the most accurate prediction models among the four tested SDMs (BIOCLIM, SVM, DOMAIN, and MAXENT). Collectively, these studies indicate the superior predictive accuracy and stability of MAHAL, RF, MAXENT, SVM over other models, including the BIOCLIM and DOMAIN models study here.

Different SDMs are differentially sensitive to various environmental variables, thereby affecting stability. Alternatively, models less sensitive to these changes should have greater stability [57]. We speculate that the superior stability and prediction performance of these four models (BIOCLIM, SVM, DOMAIN, and MAXENT) are due to methodological advances in machine learning, mathematical modeling, and the statistical tools employed (Table S2). Several recent SDMs that consider more recent ecological findings and incorporate the improved mathematical modeling techniques, machine learning algorithms, and more robust statistical tools demonstrate greater predictive accuracy than earlier SDMs [9], [34], [41], [43], [58]. For example, MAXENT has some inherent advantages including: (1) It can consider interactions between environmental variables, (2) It has efficient deterministic algorithms which can be benefit to predict species’ optimal probability distribution, and (3) It can avoid over-fitting [41]. The principle of MAXENT is to satisfy all known conditions without making subjective assumptions. When we use MAXENT to forecast the probability distribution of a random event, the probability distribution is more uniform and the stability is higher [41].

Of course, many other variables, such as species rarity, sample size, spatial scale, size of the species' geographic range, the selection of environment variables, selection method for pseudo-absence data, and the autocorrelation between geography and space can affect predictive performance [15]–[17], [59]. Additional methodological improvements that may obviate potential problems such as over-fitting and over-dispersion include: (1) assessing the influence of different scales according to species dispersal capacity, behavior, and the extent of the study area, and (2) applying enhanced frameworks by better reflecting observed species population trends and distributions for assessing uncertainties and errors in SDMs. Though some studies have also confirmed that MAHAL, RF, MAXENT, and SVM are superior to BIOCLIM and DOMAIN [16], [17], [34], [35], [58], [60], whether the prediction performances are also superior for other species or similar species in different geographic regions requires further study.

With the development of SDMs, some dynamic models can also reflect the environmental dynamic change and biological dynamic response, and they are helpful to truly reflect species potential dynamic distribution. We therefore suggest that future studies should develop more sophisticated dynamic models by incorporating some dynamic parameters (e.g. dynamic environmental variables, time of development events, growth rates, species migration ability, competitive interactions, or species sensitivity to climate), which are known to affect species potential distribution patterns but are often ignored in traditional static SDMs.

## Supporting Information

Figure S1 **Pearson’s correlation coefficients (** ***R*** **s) of 26 environmental variables. Note: Dissimilarity = 1- Pearson’s correlation coefficients (** ***Rs*** **).**
(DOC)

Table S1 **Pearson’s correlation coefficients (** ***R*** **s) between 26 environmental variables.**
(XLS)

Table S2 **Modeling data, advantages and disadvantages.**
(DOC)

Table S3 **The AUC and Kappa values for each species in six SDMs (BIOCLIM, DOMAIN, MAHAL, RF,** **MAXENT, and SVM).**
(DOC)

References S1 (DOC)
