# Supplementary material for: The Predictive Performance and Stability of Six Species Distribution Models
Source: PLoS One. 2014 Nov 10;9(11):e112764. doi: 10.1371/journal.pone.0112764 (PMC4226630; doi:10.1371/journal.pone.0112764)

**Appendix Figure S1. Pearson’s correlation coefficients (*R*s) of 26 environmental variables. Note: Dissimilarity=1-Pearson’s correlation coefficients (*Rs*).**


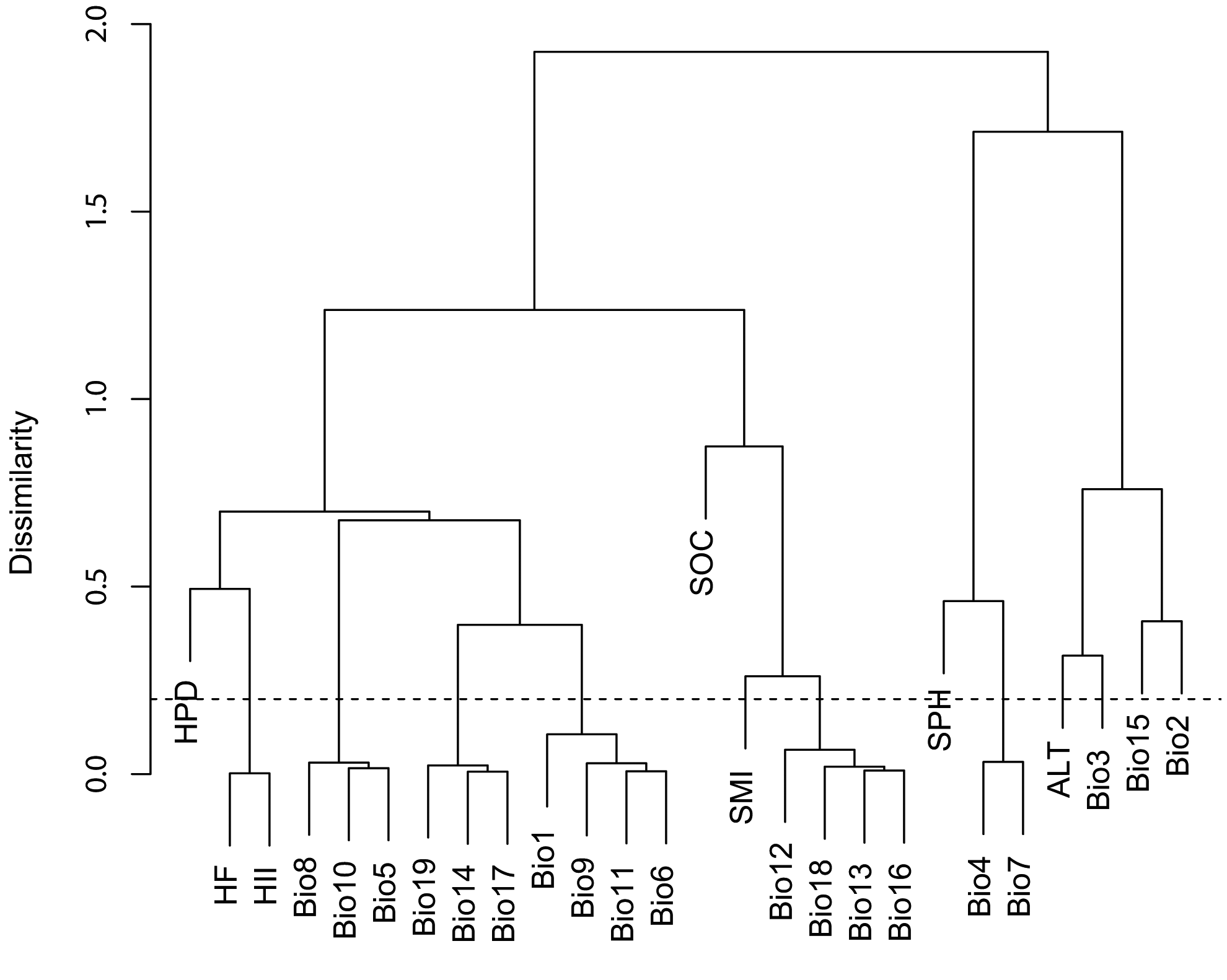

Supplement: Figure S1 — Pearson’s correlation coefficients ( R s) of 26 environmental variables. Note: Dissimilarity = 1- Pearson’s correlation coefficients ( Rs ). (DOC) [file pone.0112764.s001.doc]
