# Supplementary material for: The Predictive Performance and Stability of Six Species Distribution Models
Source: PLoS One. 2014 Nov 10;9(11):e112764. doi: 10.1371/journal.pone.0112764 (PMC4226630; doi:10.1371/journal.pone.0112764)
Supplement: Table S2 — Modeling data, advantages and disadvantages. (DOC) [file pone.0112764.s003.doc]

**Table S2. Modeling data, advantages and disadvantages.** (DOC)

| **Method** | **Data type for model fitting (training)** | **Data type for model evaluation (testing)** | **Advantages** | **Disadvantages** |
| --- | --- | --- | --- | --- |
| BIOCLIM | Presence-only | Presence and pseudo-absence | Ability to cope with ‘presence only’ data;  intuitive simplicity, clear theoretical basis (niche theory);  applicability to different taxa;  straightforwardness of operation [1]. | Inability to cope with correlations and interactions among the climatic factors;  assignment of equal suitability for all climatic combinations within the boundaries of the climatic envelope;  sensitivity to outliers [1];  gives equal weight to all environmental predictors [2]. |
| DOMAIN | Presence-only | Presence and pseudo-absence | A useful complementary alternative for general potential mapping;  it is particularly well suited to applications where available site location records or environmental data are limited [3]. | An intermediate predictive performance [4]. |
| MAHAL | Presence-only | Presence and pseudo-absence | It is calculated based on the covariance matrix of the environmental variables and, therefore, more directly reflects patterns of correlations between the environmental factors [1,4]. | Due to limitations of the algorithm mechanism, MAHAL can’t be used under such conditions: samples number are less than environment variables number or some special data set cause inverse covariance matrix not exists [5] |
| RF | Presence and absence | Presence and pseudo-absence | RF is a classification and regression tree (CART) based bootstrap method that corrects many of the known issues in CART, such as over-fitting [6];  RF has great predictive performance [7];  It is one of the most accurate learning algorithms available. For many data sets, it produces a highly accurate classifier.  It runs efficiently on large databases [8]. | For data including categorical variables with different number of levels, RF is biased in favor of those attributes with more levels. Therefore, the variable importance scores from RF are not reliable for this type of data [8]. |
| MAXENT | Presence-only ^a^ | Presence and pseudo-absence | It requires only presence data, together with environmental information for the whole study area. It can utilize both continuous and categorical data, and can incorporate interactions between different variables. Efficient deterministic algorithms have been developed that are guaranteed to converge to the optimal (maximum entropy) probability distribution. The MAXENT probability distribution has a concise mathematical definition, and is therefore amenable to analysis. Over-fitting can be avoided by using ℓ1-regularization and so on [9]. | It uses an exponential model for probabilities, which is not inherently bounded above and can give very large predicted values for environmental conditions outside the range present in the study area. Extra care is therefore needed when extrapolating to another study area or to future or past climatic conditions (for example, feature values outside the range of values in the study area should be “clamped”, or reset to the appropriate upper or lower bound) [9]. |
| SVM | Presence and absence | Presence and pseudo-absence | SVM has good generalization ability;  SVM also stands out for their robustness to high dimensional data [10]. | The difficulty of interpreting the generated model and their sensibility to a proper parameter tuning [10]. |

^a^ Use randomly sampled ‘‘background’’ sites, but not models where both presence and absence sites are used to fit, consider as PO model
