# Supplementary material for: The Predictive Performance and Stability of Six Species Distribution Models
Source: PLoS One. 2014 Nov 10;9(11):e112764. doi: 10.1371/journal.pone.0112764 (PMC4226630; doi:10.1371/journal.pone.0112764)
Supplement: Table S3 — The AUC and Kappa values for each species in six SDMs (BIOCLIM, DOMAIN, MAHAL, RF, MAXENT, and SVM). (DOC) [file pone.0112764.s004.doc]

Table S3 The AUC and Kappa values for each species in six SDMs (BIOCLIM, DOMAIN, MAHAL, RF, MAXENT, and SVM)**.**

| Species | Performance criteria | BIOCLIM | DOMAIN | MAHAL | MAXENT | RF | SVM | Mean |
| --- | --- | --- | --- | --- | --- | --- | --- | --- |
| *Betula platyphylla* | AUC | 0.946 | 0.946 | 0.966 | 0.971 | 0.971 | 0.966 | 0.961 |
|  | Kappa | 0.826 | 0.797 | 0.856 | 0.869 | 0.880 | 0.874 | 0.850 |
| *Quercus wutaishanica* | AUC | 0.941 | 0.953 | 0.981 | 0.983 | 0.986 | 0.981 | 0.971 |
|  | Kappa | 0.856 | 0.799 | 0.904 | 0.892 | 0.910 | 0.884 | 0.874 |
| *Pinus massoniana* | AUC | 0.903 | 0.922 | 0.941 | 0.951 | 0.951 | 0.940 | 0.935 |
|  | Kappa | 0.804 | 0.807 | 0.818 | 0.845 | 0.850 | 0.837 | 0.827 |
| *Quercus mongolica* | AUC | 0.949 | 0.965 | 0.977 | 0.980 | 0.981 | 0.977 | 0.972 |
|  | Kappa | 0.865 | 0.864 | 0.911 | 0.911 | 0.929 | 0.924 | 0.901 |
| *Quercus variabilis* | AUC | 0.968 | 0.979 | 0.989 | 0.989 | 0.990 | 0.987 | 0.984 |
|  | Kappa | 0.898 | 0.880 | 0.945 | 0.929 | 0.941 | 0.935 | 0.921 |
