# Supplementary material for: The Predictive Performance and Stability of Six Species Distribution Models
Source: PLoS One. 2014 Nov 10;9(11):e112764. doi: 10.1371/journal.pone.0112764 (PMC4226630; doi:10.1371/journal.pone.0112764)
Supplement: References S1 — (DOC) [file pone.0112764.s005.doc]

References S1. (DOC)

1. Farber O, Kadmon R (2003) Assessment of alternative approaches for bioclimatic modeling with special emphasis on the Mahalanobis distance. Ecol Model 160: 115–130.

2. Parra JL, Graham CC, Freile JF (2004) Evaluating alternative data sets for ecological niche models of birds in the Andes. Ecography 27: 350–360.

3. Carpenter G, Gillison A, Winter J (1993) DOMAIN: a flexible modelling procedure for mapping potential distributions of plants and animals. Biodivers Conserv 2: 667–680.

4. Tsoar A, Allouche O, Steinitz O, Rotem D, Kadmon R (2007) A comparative evaluation of presence-only methods for modelling species distribution. Divers Distrib 13: 397–405.

5. Mahalanobis PC (1936) On the generalized distance in statistics. Proc Natl Inst Sci Calcutta 2: 49–55.

6. Breiman L (2001) Random forests. Mach Learn 45: 5–32.

7. Lawler JJ, White D, Neilson RP, Blaustein AR (2006) Predicting climate-induced range shifts: model differences and model reliability. Glob Change Biol 12: 1568–1584.

8. Predrag R (n.d.) Random Forest. Available: home.etf.rs/~vm/os/dmsw/Random%20Forest.pptx‎.

9. Phillips SJ, Anderson RP, Schapire RE (2006) Maximum entropy modeling of species geographic distributions. Ecol Model 190: 231–259.

10. Lorena AC, Jacintho LFO, Siqueira MF, Giovanni RD, Lohmann LG, et al. (2011) Comparing machine learning classifiers in potential distribution modelling. Expert Syst Appl 38: 5268 – 5275.
